# Removing Deer Mice from Buildings and the Risk for Human Exposure to Sin Nombre Virus

**DOI:** 10.3201/eid0903.020470

**Published:** 2003-03

**Authors:** Richard J. Douglass, Amy J. Kuenzi, Courtney Y. Williams, Samuel J. Douglass, James N. Mills

**Affiliations:** * Montana Tech of the University of Montana, Butte, Montana, USA; † University of California School of Veterinary Medicine, Davis, California, USA; ‡ Clark’s Honors College, University of Oregon, Eugene, Oregon, USA; § Centers for Disease Control and Prevention, Atlanta, Georgia, USA

## Abstract

Trapping and removing deer mice from ranch buildings resulted in an increased number of mice, including Sin Nombre virus antibody–positive mice, entering ranch buildings. Mouse removal without mouse proofing will not reduce and may even increase human exposure to Sin Nombre hantavirus.

Sin Nombre virus (SNV), carried by the deer mouse (*Peromyscus maniculatus*) is the etiologic agent of hantavirus pulmonary syndrome (*1*). Most cases of this syndrome occur after exposure to deer mice in peridomestic settings (*2*); the prevalence of antibody to SNV may be higher in peridomestic populations than in sylvan populations (*3*). In addition, some rodent species move readily between sylvan and peridomestic settings (*3*). Rodent removal combined with mouse proofing of human dwellings eliminates rodent-human contact in treated structures (*4*); however, removal without mouse proofing may not be effective (*4*) because removal may induce mouse immigration into the area (*5*). Additionally, if mice are trapped alive and released outdoors even at some distance, they will often return (*6*). Our study consisted of two experiments designed to determine the efficacy of reducing human exposure to rodents by removing deer mice from outbuildings that were not mouse proofed.

## The Study

To determine how removal from outbuildings affects abundance of mice in structures, two removal experiments were conducted in Montana, where deer mice are commonly found in buildings. For both experiments, we followed the handling protocols as described (*7*), except that we did not anesthetize the mice. We collected data as described (*8*).

In experiment 1, we trapped live deer mice in 16 ranch-yard outbuildings (peridomestic area), as well as nearby sylvan habitats, for 3 nights each week from mid-June to mid-August, 1999. The peridomestic area, about 1 ha in size, contained buildings and corrals. We trapped mice only in buildings in the peridomestic area. Four of the sites were designated “removal buildings,” and all animals captured from these buildings were euthanized. Captured mice from the remaining 12 “control buildings” were marked and released. We set a total of 100 traps in buildings; the number of traps per building was determined by building size (16–40 m^2^ with an average of one trap/4 m^2^). During all trapping periods, the number of traps set was always more than the number of animals captured in every building. In the sylvan area (1.1 ha), we placed 100 traps in four parallel rows. Sets of two rows were placed on either side of the ranch yard; traps were located 20–100 m from the nearest building. We marked and released animals for 7 days during study week 1, then we removed them for weeks 2–8.

In experiment 2, we examined the effect of deer mouse removal on SNV-antibody prevalence in buildings. The site for this experiment was approximately 6 km from the site of experiment 1. On the experiment 2 site, we had conducted extensive work from November 1996 to April 1999; the site included three buildings as previously described (*3*). Two buildings were designated removal buildings and the third a control building. For 11 weeks in fall 1999 and 5 weeks in spring 2001, we collected blood from all removed and control animals (control animals at first capture only). In fall 1999, we trapped and removed mice daily from removal buildings during week 1, for 5 days during week 2, and for 3 days weekly during weeks 3–11. In spring 2001, animals were removed or marked and released (control building) for 3 days each week for 5 weeks.

In experiment 1, we captured a total of 133 deer mice in the sylvan (38 mice) and peridomestic (95 mice) areas (Table). We removed 52 deer mice from the four removal buildings. Immigrant mice quickly replaced resident deer mice removed from these buildings. This replacement resulted in a higher average number of deer mice captured in the four buildings from which we removed animals (13.8 individual mice/building; 95% confidence interval [CI] 7.6 to 20.0) than in buildings from which no mice were removed (5.8 mice/building; 95% CI 3.6 to 8.0). Of the deer mice previously captured in the sylvan area (20–100 m away), 7.9% immigrated into the removal buildings, and 16.8% moved from building to building (Table).

**Table:** Deer mice removed from buildings and sources of immigrating deer mice on a ranch, southwestern Montana, 1999

Capture location		Total	Removed	Moved^a^	Source of post-removal immigration	
Sylvan	Buildings				Sylvan to building	Building to building
38	95	133	52	19	7.9% (n=3)^b^	16.8% (n=6)^b^
					5.7%^c^	30.1%^c^

^a^No. of deer mice that moved from previous capture areas (source) to removal site. ^b^Percentage of marked source population. ^c^Percentage of total deer mice removed.

In experiment 2, a total of 54 deer mice were captured from all three buildings. Thirty deer mice were taken from the two removal buildings in 1999 and six in 2001. In the 1999 sample, more deer mice were captured in each of the two removal buildings than had been captured in the same buildings during either of the previous two fall seasons (*3*). The number of deer mice that occupied the control building was similar to the number reported for the previous two fall seasons. Although the spring 2001 sample was too small for statistical analysis, five deer mice were captured in one removal building and two in the control building. One deer mouse was captured from the other removal building (Figure). Notably, the two deer mice captured in the control building continued to occupy the buildings for all trapping periods. During the initial removal (fall 1999), none of 15 captured deer mice had detectable antibody to SNV. However, subsequent to removal, three immigrant mice were found to be antibody-positive when first captured, while deer mice occupying the control building remained antibody-negative (Figure). At various times during previous years of sampling (*3*) in these buildings, the control building for this study contained antibody-positive mice, as did the removal buildings. During the preceding two falls, no antibody-positive deer mice had been captured in the removal building in which antibody-positive mice were captured in the experiment.

**Figure:**
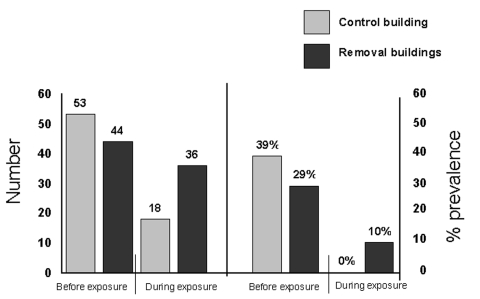
Number and Sin Nombre virus–antibody prevalence in deer mice found in removal and control buildings, Montana. Data combined from previous study (left side, [3]) and from experiments conducted in fall 1999 and spring 2001 (right side).

## Conclusions

Our data show that removing deer mice did not reduce their population numbers in any building. Outbuildings are normal habitats for deer mice in Montana, and all of the buildings in experiment 2 had resident deer mice for 3 years before this study (*3*). In sylvan habitats when resident deer mice are removed, immigrant mice quickly replace them (*9*) and often travel long distances to do so (*10*). Entire sylvan population can be replaced in 2 weeks (*5*).

Under certain circumstances, removal could substantially reduce the number of mice. The number of dispersing deer mice is linearly related to the density of the source population, and the rate of dispersal is correlated with the rate of increase in the source population (*11*). Extremely large fluctuations have been documented in Montana deer mouse populations (*8*). These fluctuations affect dispersal rates and entrance into buildings.

Outbuildings, though originally colonized from sylvan populations of deer mice, also act as sources (Table). The total peridomestic area occupied by buildings in experiment 1 (approximately 1 ha) is only slightly larger than the home ranges of some deer mice (*10*). Removing animals from 4 of the 16 buildings may have rearranged territories within a small area without creating the large vacant habitats reported in previous removal studies (*9*,*5*,*11*). Removal of deer mice from all buildings within the ranch yard might have resulted in migration from surrounding sylvan habitats larger than those identified in this study.

Removing animals from outbuildings also creates a constant turnover in a building’s deer mouse population; thus, more deer mice would be captured in a building over time than if mice had not been removed. This constant turnover increases the probability that an antibody-positive mouse will enter the building. The entrance of antibody-positive mice into the removal building in experiment 2 is consistent with this concept. Removing animals from some but not all buildings initiates movement of mice from other buildings. Such removal rearranges local territories and may alter the proportion of SNV-infected deer mice, which in turn, may alter the probability of human exposure to SNV.

In summary, our study showed that removal of deer mice from non–rodent-proofed ranch buildings did not reduce rodent infestation of these buildings. An increase in the number of deer mice occurred in most buildings from which mice had been removed. In three instances, SNV antibody-negative mice in the buildings were replaced after their removal by antibody-positive mice. These results suggest that rural homeowners who trap deer mice in homes or outbuildings without first attempting to seal the structures against renewed infestation are not decreasing their risk of exposure to SNV. Detailed procedures for rodent proofing have been described (*12*), as well as procedures for safe trapping and handling of captured mice (*7*).
